# Development and validation of a patient-reported outcome measure for stroke patients

**DOI:** 10.1186/s12955-015-0246-0

**Published:** 2015-05-08

**Authors:** Yanhong Luo, Jie Yang, Yanbo Zhang

**Affiliations:** Department of Health Statistics, School of Public Health, Shanxi Medical University, 56 South XinJian Road, Taiyuan, Shanxi Province 030001 People’s Republic of China

**Keywords:** Stroke, Patient-reported outcome, Item response theory, Classical test theory, Confirmatory factor analysis, Reliability, Validity, Measurement

## Abstract

**Background:**

Family support and patient satisfaction with treatment are crucial for aiding in the recovery from stroke. However, current validated stroke-specific questionnaires may not adequately capture the impact of these two variables on patients undergoing clinical trials of new drugs. Therefore, the aim of this study was to develop and evaluate a new stroke patient-reported outcome measure (Stroke-PROM) instrument for capturing more comprehensive effects of stroke on patients participating in clinical trials of new drugs.

**Methods:**

A conceptual framework and a pool of items for the preliminary Stroke-PROM were generated by consulting the relevant literature and other questionnaires created in China and other countries, and interviewing 20 patients and 4 experts to ensure that all germane parameters were included. During the first item-selection phase, classical test theory and item response theory were applied to an initial scale completed by 133 patients with stroke. During the item-revaluation phase, classical test theory and item response theory were used again, this time with 475 patients with stroke and 104 healthy participants. During the scale assessment phase, confirmatory factor analysis was applied to the final scale of the Stroke-PROM using the same study population as in the second item-selection phase. Reliability, validity, responsiveness and feasibility of the final scale were tested.

**Results:**

The final scale of Stroke-PROM contained 46 items describing four domains (physiology, psychology, society and treatment). These four domains were subdivided into 10 subdomains. Cronbach’s α coefficients for the four domains ranged from 0.861 to 0.908. Confirmatory factor analysis supported the validity of the final scale, and the model fit index satisfied the criterion. Differences in the Stroke-PROM mean scores were significant between patients with stroke and healthy participants in nine subdomains (*P* < 0.001), indicating that the scale showed good responsiveness.

**Conclusions:**

The Stroke-PROM is a patient-reported outcome multidimensional questionnaire developed especially for clinical trials of new drugs and is focused on issues of family support and patient satisfaction with treatment. Extensive data analyses supported the validity, reliability and responsiveness of the Stroke-PROM.

**Electronic supplementary material:**

The online version of this article (doi:10.1186/s12955-015-0246-0) contains supplementary material, which is available to authorized users.

## Background

Stroke is the second leading cause of mortality worldwide [1], and stroke survivors are often severely disabled for the rest of their lives [2]. More than 85% of strokes occur in developing countries [3]. Epidemiological surveys have shown that there are 150–200 million new cases of stroke each year in China. The age-adjusted annual incidence rate of stroke is 116–219 per 100,000 people, and the annual mortality rate from stroke is 58–142 per 100,000 people [4].

Stroke has considerable adverse physical and psychological impacts on patients over time [5,6]. For the diagnosis and treatment of stroke and its sequelae, therefore, purely objective indicators do not accurately measure the multifaceted impact of stroke on patients. Assessment of the effects of treatment on any individual patient should include the patient’s own evaluation of therapy, or patient-reported outcome (PRO) [7]. A PRO is any report of the status of a patient’s health condition that comes directly from the patient, without interpretation of the patient’s response by a clinician or anyone else [8,9].

In recent years, multiple measures, including generic and disease-specific measures, have been used to assess outcomes of patients with stroke. Generic instruments are useful for comparing quality of life impact in populations with different diseases; however, disease-specific tools are generally more responsive and sensitive to disease-specific issues and are therefore more appropriate for clinical trials in which specific therapeutic interventions are being evaluated [10,11]. Although PRO tools developed specifically for stroke do exist (e.g., Newcastle Stroke-Specific Quality of Life Measure; Stroke and Aphasia Quality of Life Scale-39 item version; Stroke Impact Scale version 2.0), a review of these instruments yielded no measure that captures PRO associated with family support and patient satisfaction with treatment, two particularly significant issues for many stroke survivors [12-15]. Given the absence of stroke-specific measures in the subdomains of family support and treatment satisfaction, the development is necessary of a more comprehensive multidimensional scale that evaluates all facets of the health status in patients with stroke.

Therefore, the aim of this study was to develop an understandable, reliable and valid PRO measure for patients with stroke that captures valuable data from the patient’s viewpoint. This article reports on the development of the initial pool of items, selection of the final item set, and evaluation of a new stroke patient-reported outcome measure (Stroke-PROM).

## Methods

### Ethics statement

The study protocol and the Stroke-PROM were reviewed and approved by the Medical Ethics Committee of Shanxi Medical University. Participants signed informed consent forms prior to study participation, and all were compensated for their time.

### Study population and design

Patients were enrolled from nine different hospitals, communities, and rural areas in Shanxi province in China. Clinical investigators at all study sites recruited participants using the International Classification of Diseases, Ninth Revision, Clinical Modification (ICD-9-CM). Patients participating in this study were diagnosed with stroke by a physician and were not in the acute phase of stroke. The severity of poststroke sequelae in these patients varied from mild to severe. Individuals with tetraplegia, psychosis, or serious comorbidities (e.g., cancer) were excluded. Control participants were recruited from lists of patients who did not have cerebral vascular disease, cancer, or mental illness. Investigators helped patients with severe visual impairments fill in the questionnaires according to the patients’ verbal responses to items.

Ten patients with stroke were interviewed to identify potential items for use in the questionnaire. Five patients with stroke, three physician experts in stroke and one psychometric expert were interviewed for item revision and refinement to ensure that all items were appropriate and relevant. Five stroke patients were interviewed to evaluate their comprehension of each item. For the first item pool reduction, 135 patients with stroke were recruited from nine different hospitals, communities, and rural areas in Shanxi province; valid data from 133 participants were collected. For the item-revaluation phase and the validation phase of the Stroke-PROM, 485 patients with stroke and 110 controls from the same nine geographical regions were recruited, but only 475 and 104, respectively, were available to participate in the study. There was no overlap in the participants who contributed to the first and second item-reduction processes [16,17].

### Development of the Stroke-PROM

**Figure 1:**
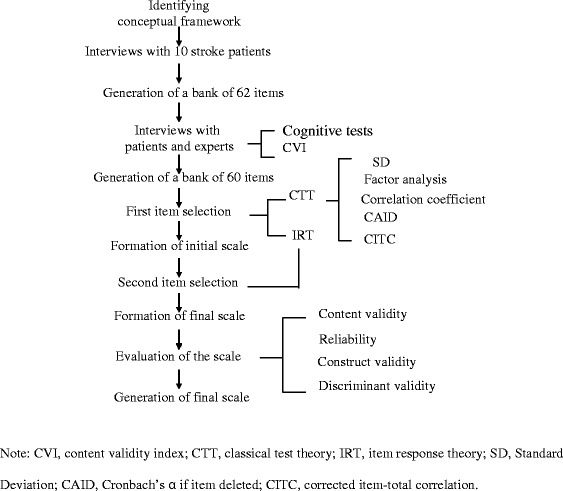
Flowchart of the Stroke-PROM developmental process. Note: CVI, content validity index; CTT, classical test theory; IRT, item response theory; SD, Standard Deviation; CAID, Cronbach’s α if item deleted; CITC, corrected item-total correlation.

The Stroke-PROM was developed in four phases: (1) conceptual framework construction and preliminary item generation; (2) formation of the initial scale by the first item-selection process; (3) formation of the final scale by an item-revaluation process based on the second item-selection process; and (4) validation of the Stroke-PROM. Phase 1 involved a qualitative analysis, whereas the other three phases used quantitative analyses. A flowchart of this four-phase developmental process is shown in Figure 1.

### Identifying the conceptual framework and preliminary item content

**Figure 2:**
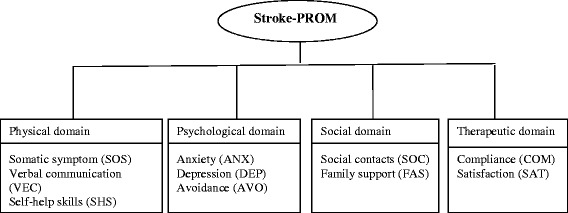
Conceptual framework of the Stroke-PROM.

A comprehensive review of existing stroke questionnaires was performed to identify an appropriate conceptual framework (see Figure 2). Four domains and 10 subdomains were generated. In-depth open-ended interviews of 10 stroke patients (5 men and 5 women; ages: ≤45, n = 2; 45–65, n = 5; ≥65, n = 3) were conducted to identify potential items for the Stroke-PROM using the selected conceptual framework. Patients were interviewed about their symptoms, their main psychological burden, the effects of stroke on them and their families, and their evaluations of the therapeutic effect and medical workers. As a result, a bank of 62 potential items was generated. Four chief physicians and five patients (3 men and 2 women; ages: ≤45, n = 1; 45–65, n = 2; ≥65, n = 2; education: high school degrees or above), all of whom were recruited from the First Hospital of Shanxi Medical University and Second Hospital of Shanxi Medical University, participated in revising these 62 preliminary items using a content validity index (CVI) (62 items and scale structure described in Additional file 1: Appendices 1–1 and 1–2).

The CVI is widely used for quantifying content validity for scales. Item-level CVI (I-CVI) is calculated by having experts rate the relevance of each item to its own subdomain (1 = not relevant, 2 = somewhat relevant, 3 = quite relevant, 4 = highly relevant). The I-CVI of each item is defined as the number of experts offering a rating of 3 or 4, divided by the number of experts.

As an adjustment for chance agreements, the multi-rater kappa statistic (K^*^) was adopted and is described as follows [18,19]:

$$ {p}_c=\left[\frac{n!}{A!\left(n-A\right)!}\right]\times {0.5}^n $$

where *P*_*c*_ is the probability of chance agreement, *n* is the number of experts, and *A* is the number approving with good relevance. K^*^ was calculated using the I-CVI and the probability of chance agreement as follows:

$$ {k}^{*}=\frac{I-CVI-{P}_c}{1-{P}_c} $$

Each item on the scale was then rated as “fair,” “good,” or “excellent,” based on the following rating criteria: fair, K^*^ = 0.40–0.59; good, K^*^ = 0.60–0.74; excellent, K^*^ > 0 .74. Any item that received a “fair” rating was deleted.

Five participants with stroke (2 men, 3 women; ages: ≤45, n = 1; 45–65, n = 2; ≥65, n = 2) were interviewed to evaluate their comprehension of each item. Items that were ambiguous, misunderstood, or rarely answered were reworded. The preliminary scale was developed after modifying the item pool based on the suggestions of the physician experts, psychometric expert and patients as well as on the outcomes of the comprehension tests for patients. In the end, the preliminary tool included 4 domains, 11 subdomains, and 60 items.

### Formation of the initial scale

One hundred thirty-three patients were told that the aim of the questionnaire was to measure how much their stroke had affected them. For each issue presented in an item, patients responded using a five-point Likert scale to reflect how often they experienced the issue, where 0 = never, 1 = occasionally, 2 = about half of the time, 3 = often, and 4 = always. Scores of positively worded items were recoded as the original score plus 1, whereas scores of negatively worded items were recoded as 5 minus the original response. This recoding produced a score range for each item of 1 to 5, with a higher score reflecting a more positive PRO.

The item-reduction processes of the preliminary scale were based on both classical test theory (CTT) (e.g., discrete trend, factor analysis, correlation coefficient, Cronbach’s α if item deleted [CAID] values, and corrected item-total correlation [CITC]) and item response theory (IRT). CTT was used to reduce the number of items of the Stroke-PROM in the first four of the following steps, and IRT was used in the fifth step.

In step 1, the standard deviation in the score for every item was calculated. A low standard deviation indicates a low degree of differentiation and should be removed; thus, those items with a low standard deviation (<0.96) were deleted in this study.

In step 2, a principal component factor analysis with varimax rotation aided in item reduction. The value for the Kaiser–Meyer–Olkin measure of sampling adequacy was >0.5 [17]. Items with low factor loading (<0.4) or items with factor loading close to other factors was considered for removal.

In step 3, an item was considered for deletion when the Pearson correlation coefficient between the item and its own subdomain was <0.6, which indicated that the item did not represent the subdomain well.

In step 4, the internal consistency of items was evaluated using the CITC and CAID values. An item was considered to have highly contributed to the measured construct when the CITC value was more than 0.45. The CAID value also determines which item highly contributes to the reliability of the Stroke-PROM. An increase in the CAID value indicates that the items poorly contribute to Cronbach’s α value and should be deleted. Therefore, an item was deleted in the present study when the CITC value was <0.45 and the CAID value increased [20-22].

In step 5, IRT was applied to reduce the number of items in the Stroke-PROM. Each item’s parameters of discrimination (α) and difficulty (b) were estimated. Generally, items with discrimination values <0.4 should be deleted. The value of the four degrees of difficulty (*b*_1_, *b*_2_, *b*_3_, *b*_4_) ranged from −3 to 3. Items with degrees of difficulty (*b*_1_, *b*_2_, *b*_3_, *b*_4_) values outside the range of −3 to 3 should be considered for removal [23].

Both the statistical results and clinical relevance of items were also taken into account prior to an item’s deletion. The resulting initial scale resulted from the removal of items from the preliminary scale.

### Formation and validation of the final scale

Thus, an initial scale was generated following the evaluation and selection of items from the preliminary scale. To ensure the reliability and validity of each item included in this initial Stroke-PROM, the items were re-evaluated based on a second item-selection of the initial scale. The CTT and IRT were applied once again to re-evaluate the items in the initial Stroke-PROM using the data gathered from 475 stroke patients, generating the final scale. The final Stroke-PROM tool was then evaluated for validity, reliability, and responsiveness using the data obtained from these 475 stroke patients as well as 104 control participants.

### Content validity

Content validity was achieved by referring to relevant literature, consulting questionnaires from China and other countries, interviewing 10 patients to identify potential items, and consulting with 5 patients, 3 physician experts and 1 psychometric expert for item revision and refinement to ensure that all items were appropriate and relevant. Content validity was confirmed using the CVI.

### Construct validity

Confirmatory factor analysis with the index of model fit was performed to investigate the factor structure of the scale [23]. The model indicates a good fit when the goodness-of-fit index (GFI), normed fit index (NFI), non-normed fit index (NNFI), incremental fit index (IFI) and comparative fit index (CFI) are all >0.9, and the root mean square residual (RMR) is <0.09. GFI, RMR, NFI and CFI range from 0 to 1.

### Reliability

Cronbach’s α coefficients for the four domains and the total scale were calculated to measure the internal consistency of the Stroke-PROM. Generally a Cronbach’s α coefficient ≥ 0.7 indicates an acceptable level of internal consistency.

### Discriminant validity

The modified Rankin Scale, a frequently used scale for measuring the degree of disability and dependence in the daily activities of people who have had a stroke, was used as the stroke outcome measure in the present study. This ordered scale ranges from 0 (no symptoms) to 5 (severe disability). Discriminant validity was assessed by comparing the mean scores for every subdomain of the Stroke-PROM among healthy participants with those among groups of stroke patients as defined by the Rankin scale, except for the subdomain of treatment. The comparison of means was performed using analysis of variance, with the significance level set at p < 0.05. The rejection of the null hypothesis would indicate that the scale has the ability to differentiate between healthy controls and stroke patients with varying degrees of disability and dependence as defined by the modified Rankin scale.

### Feasibility

The feasibility of the Stroke-PROM tool was evaluated by examining the response rate, completion rate and response time to completion. Response and return rates above 95% were deemed adequate, and completion times of 8 to 13 minutes were considered acceptable.

### Data analysis software

Data analyses were conducted using SPSS 13.0, Multilog 7.03 and LISREL 8.70 software.

## Results

### Participant characteristics

Tables 1 and 2 show the characteristics of the 133 patients with stroke who completed the preliminary scale and of the 475 stroke patients and 104 control participants who completed the initial scale.

**Table 1:** **Demographic characteristics of 133 patients with stroke in the first item-selection phase**

**Characteristics**	**Stroke patients**
**Sex (n (%))**	
Male	78 (58.4)
Female	55 (41.4)
**Age (n (%))**	
≤45	9 (6.8)
45–65	69 (51.9)
≥65	55 (41.4)
**Marital status (n (%))**	
Married	114 (85.7)
Unmarried, divorce or widowed	19 (14.3)
**Highest education level completed (n (%))**	
Primary school or lower	53 (39.8)
Junior high school	45 (33.8)
Senior high school	19 (14.3)
College or higher	16 (12.0)

**Table 2:** **Demographic characteristics of 475 stroke patients and 104 controls in the second item-selection phase and the Stroke-PROM validation phase**

**Characteristics**	**Stroke patients**	**Controls**	***χ*** ^**2**^	***P***
**Sex (n (%))**				
Male	283 (59.6)	49 (47.1)	5.418	0.020
Female	192 (40.4)	55 (52.9)		
**Age (n (%))**				
≤45	55 (11.6)	24 (23.1)	13.626	0.001
45–65	230 (48.4)	54 (51.9)		
≥65	190 (40.0)	26 (25.0)		
**Marital status (n (%))**				
Married	398 (83.8)	86 (82.7)	0.075	0.784
Unmarried, divorced or widowed	77 (16.2)	18 (17.3)		
**Highest education level completed (n (%))**				
Primary school or lower	183 (38.5)	43 (41.3)	2.870	0.412
Junior high school	144 (30.3)	37 (35.6)		
Senior high school	88 (18.5)	15 (14.4)		
College or higher	60 (12.6)	9 (8.7)		

The demographic characteristics of the participants shown in Tables 1 and 2 indicated that the stroke sample population consisted of more men than women, more than 75% of all participants were over 45 years of age, and more than 80% were married. Additionally, approximately 70% of all participants had junior high school education or less. Table 2 shows that the proportion of males with stroke was a little higher than that of healthy males, and that among participants over 65 years old, the proportion of stroke patients was slightly higher than that of healthy participants. The average length of time since stroke diagnosis was approximately 6.3 months and 7.2 months for patients in the first item pool reduction phase, and the revaluation and validation phases, respectively.

### Item generation

Four domains, 10 subdomains and a pool of 62 items were generated for the Stroke-PROM based on consulting relevant literature, examining other questionnaires, and interviewing 10 patients to ensure that all germane topics were included. The items and construction of the Stroke-PROM tool are described in Additional file 1: Appendices 1–1 and 1–2.

Four chief physicians and five patients (distinguished by different letters of the alphabet) who had attained a high school degree or above participated in the revision of the 62 items of the Stroke-PROM by rating the items according to the CVI (see Table 3).

**Table 3:** **Content validity index based on grade of patients and experts of preliminary Stroke-PROM**

**Item**	**Rating by experts**				**Rating by patients**					**Number giving rating of 3 or 4**	**I-CVI**	**P**_**C**_	**K** ^*^	**Evaluation**
	**A**	**B**	**C**	**D**	**A**	**B**	**C**	**D**	**E**					
PHD 1	3	4	4	4	3	4	4	4	3	9	1.00	0.002	1.00	excellent
PHD 2	3	4	4	3	3	4	4	4	3	9	1.00	0.002	1.00	excellent
PHD 3	2	3	3	2	2	3	3	4	2	5	0.56	0.246	0.42	fair
PHD 4	4	4	4	4	4	4	4	4	4	9	1.00	0.002	1.00	excellent
PHD 5	2	2	2	1	2	3	3	2	2	2	0.22	0.070	0.16	fair
PHD 6	2	3	2	2	2	2	3	2	1	2	0.22	0.070	0.16	fair
PHD 7	3	4	4	3	4	4	4	4	3	9	1.00	0.002	1.00	excellent
PHD 8	3	4	4	4	3	4	4	4	4	9	1.00	0.002	1.00	excellent
PHD 9	2	2	3	3	2	2	3	3	1	4	0.44	0.246	0.26	fair
PHD 10	1	2	1	2	2	2	3	2	2	1	0.11	0.018	0.09	fair
PHD 11	1	2	1	1	2	2	3	2	1	1	0.11	0.018	0.09	fair
PHD 12	3	4	3	4	4	4	4	3	2	8	0.89	0.014	0.89	excellent
PHD 13	3	2	3	4	3	4	3	3	2	7	0.78	0.070	0.76	excellent
PHD 14	3	4	4	3	3	4	3	3	2	8	0.89	0.014	0.89	excellent
PHD 15	3	4	3	3	3	3	4	4	4	9	1.00	0.002	1.00	excellent
PHD 16	3	3	2	3	2	2	3	4	4	6	0.67	0.164	0.61	good
PHD 17	3	4	4	4	4	4	4	3	4	9	1.00	0.002	1.00	excellent
PHD 18	3	4	2	4	3	3	4	2	4	7	0.78	0.070	0.76	excellent
PHD 19	3	4	4	4	4	4	4	4	4	9	1.00	0.002	1.00	excellent
PHD 20	3	3	3	4	4	4	3	4	4	9	1.00	0.002	1.00	excellent
PHD 21	3	2	4	4	4	4	4	4	3	8	0.89	0.014	0.89	excellent
PHD 22	3	3	3	3	4	3	3	4	3	9	1.00	0.002	1.00	excellent
PHD 23	3	4	4	4	4	4	4	4	3	9	1.00	0.002	1.00	excellent
PSD1	4	4	4	4	4	4	4	4	4	9	1.00	0.002	1.00	excellent
PSD2	4	4	4	4	4	4	4	4	4	9	1.00	0.002	1.00	excellent
PSD3	4	4	4	4	4	4	4	4	4	9	1.00	0.002	1.00	excellent
PSD4	4	4	4	4	4	4	4	4	4	9	1.00	0.002	1.00	excellent
PSD5	4	4	4	4	3	4	4	4	3	9	1.00	0.002	1.00	excellent
PSD6	4	4	4	4	3	4	4	4	4	9	1.00	0.002	1.00	excellent
PSD7	3	4	4	4	3	3	4	4	4	9	1.00	0.002	1.00	excellent
PSD8	3	4	4	4	3	4	4	4	4	9	1.00	0.002	1.00	excellent
PSD9	4	4	4	4	4	4	4	4	4	9	1.00	0.002	1.00	excellent
PSD10	4	4	4	4	4	4	4	4	4	9	1.00	0.002	1.00	excellent
PSD11	4	4	4	4	4	4	4	4	4	9	1.00	0.002	1.00	excellent
PSD12	4	4	4	4	4	4	4	4	4	9	1.00	0.002	1.00	excellent
PSD13	3	4	3	4	3	4	4	4	3	9	1.00	0.002	1.00	excellent
PSD14	4	4	4	4	4	4	4	4	4	9	1.00	0.002	1.00	excellent
PSD15	4	4	4	4	4	4	4	4	4	9	1.00	0.002	1.00	excellent
PSD16	4	4	4	4	4	4	4	4	4	9	1.00	0.002	1.00	excellent
PSD17	4	4	4	4	4	4	4	4	4	9	1.00	0.002	1.00	excellent
PSD18	3	4	4	2	3	4	4	3	3	8	0.89	0.014	0.89	excellent
PSD19	4	4	4	4	4	4	4	4	4	9	1.00	0.002	1.00	excellent
PSD20	4	4	4	4	4	4	4	4	4	9	1.00	0.002	1.00	excellent
SOD1	2	4	3	2	2	3	4	4	2	5	0.56	0.246	0.42	fair
SOD2	3	4	4	3	3	3	4	4	3	9	1.00	0.002	1.00	excellent
SOD3	3	4	4	4	3	4	4	4	3	9	1.00	0.002	1.00	excellent
SOD4	3	4	4	2	3	4	4	4	3	8	0.89	0.014	0.89	excellent
SOD5	4	4	4	4	4	4	4	4	4	9	1.00	0.002	1.00	excellent
SOD6	3	4	4	4	4	4	4	4	4	9	1.00	0.002	1.00	excellent
SOD7	4	4	4	4	4	4	4	4	4	9	1.00	0.002	1.00	excellent
SOD8	3	4	4	4	3	4	4	4	4	9	1.00	0.002	1.00	excellent
SOD9	4	4	4	4	4	4	4	4	4	9	1.00	0.002	1.00	excellent
SOD10	4	4	4	4	4	4	4	4	4	9	1.00	0.002	1.00	excellent
THD1	4	4	4	4	4	4	4	4	4	9	1.00	0.002	1.00	excellent
THD2	3	4	4	4	4	4	4	4	4	9	1.00	0.002	1.00	excellent
THD3	3	4	4	4	3	4	4	4	3	9	1.00	0.002	1.00	excellent
THD4	4	4	4	4	4	4	4	4	4	9	1.00	0.002	1.00	excellent
THD5	4	4	4	4	4	4	4	4	4	9	1.00	0.002	1.00	excellent
THD6	4	4	4	4	3	4	4	4	4	9	1.00	0.002	1.00	excellent
THD7	3	4	4	4	3	4	4	4	3	9	1.00	0.002	1.00	excellent
THD8	2	4	4	4	3	4	4	4	3	8	0.89	0.014	0.89	excellent
THD9	3	4	4	4	2	3	4	4	2	7	0.78	0.070	0.76	excellent

Note: Letters A to D represent individual experts; letters A to E represent individual patients; 1 = not relevant, 2 = somewhat relevant, 3 = quite relevant, 4 = highly relevant; CVI, content validity index; I-CVI, Item-level CVI; K^*^, the multi-rater kappa statistic; PHD, physical domain; PSD, psychological domain; SOD, social domain; THD, therapeutic domain.

On the basis of the CVI results, advice from patients and experts, and the clinical relevance of items, seven items (item PHD3, PHD5, PHD6, PHD9, PHD16, PSD18 and SOD1 shown in Additional file 1: Appendix 1-1) were deleted, and the subdomain of cognition was added. Items PHD10 and PHD11 shown in Additional file 1: Appendix 1-1 were retained based on the advice of patients and experts and other stroke-specific scales. These two items were assimilated into the newly added cognitive subdomain. The following five items were also added: *Have you felt any limb abnormalities [such as a burning sensation]?; Do your hands tremble when you reach for or pick up things?; Do you have trouble remembering the date?; When you see an object suddenly, do you struggle to bring its name to mind?; When others talk about your disease, do you prefer not to discuss it?* (PHD2, PHD7, PHD10, PHD11 and PSD18 shown in Additional file 1: Appendix 2-1) [13-15]. The CVI values of the five added items were calculated, the K^*^ values of the five added items were all >0.74, and the five added items were rated “excellent.”

Five stroke patients of varying educational levels were interviewed to evaluate their comprehension of each item. Items that were ambiguous, misunderstood or rarely answered were reworded using comprehension tests for patients with stroke. The preliminary scale was developed after modifying the item pool based on the advice of the experts and the outcomes of the comprehension tests. The preliminary scale included 4 domains, 11 subdomains and 60 items. The items were also reordered (see Additional file 1: Appendices 2–1 and 2–2).

### Item reduction

The two-step item selection process is described in Tables 4 and 5. This iterative process resulted in a final version that comprised 46 items within 10 subdomains. (The deletion of the compliance subdomain is explained in the next section.) Each subdomain was named according to its constituent items.

**Table 4:** **Results of the first item-selection phase using CTT and IRT**

**Item**	**SD**	**Factor loading**	**Correlation coefficient**	**CITC**	**CAID**	**IRT**					**Outcome**
						***α***	***b***_***1***_	***b***_***2***_	***b***_***3***_	***b***_***4***_	
PHD1	1.464	0.752	0.784	0.705	0.908	0.88	−1.67	−0.37	0.09	1.72	√
PHD2	1.423	0.706	0.763	0.712	0.906	1.13	−1.85	−0.39	−0.12	1.16	√
PHD3	1.446	0.799	0.811	0.77	0.900	1.30	−1.44	−0.78	−0.32	0.36	√
PHD4	1.355	0.734	0.822	0.739	0.903	1.26	−1.16	−0.10	0.61	1.97	√
PHD5	1.320	0.845	0.799	0.825	0.894	1.59	−1.78	−0.71	−0.34	0.25	√
PHD6	1.246	0.811	0.758	0.768	0.901	1.29	−2.20	−0.93	−0.22	0.77	√
PHD7	1.213	0.722	0.743	0.689	0.908	1.57	−1.90	−0.72	0.03	0.98	√
PHD8	1.439	0.781	0.835	0.628	**0.869**	0.53	**−3.23**	−1.31	−0.32	1.77	X
PHD9	1.325	0.777	0.818	0.697	0.836	1.18	−1.98	−0.70	−0.10	1.05	√
PHD10	1.266	0.801	0.802	0.777	0.804	1.29	−1.71	−0.97	−0.49	0.95	√
PHD11	1.214	0.745	0.813	0.779	0.806	1.63	−1.89	−0.89	−0.37	0.49	√
PHD12	1.395	0.473	0.657	0.515	0.694	1.37	−1.62	−0.58	−0.38	0.50	√
PHD13	1.310	0.514	0.664	0.584	0.659	1.15	−1.89	−1.11	−0.53	0.61	√
PHD14	1.520	0.489	0.793	0.478	0.718	**0.29**	**−4.94**	−2.28	−0.60	2.39	√
PHD15	1.471	0.549	0.748	0.573	0.661	**0.30**	**−5.24**	**−4.20**	−2.91	0.52	√
PHD16	1.582	0.786	0.739	0.634	0.789	0.47	−2.40	−1.77	−1.07	1.19	√
PHD17	1.500	0.779	0.740	0.676	0.782	**0.37**	**−3.83**	−2.92	−1.87	0.97	√
PHD18	**0.930**	**0.081**	**0.405**	**0.208**	**0.843**	0.90	**−4.18**	−2.86	−2.25	−1.15	X
PHD19	1.535	**0.262**	**0.495**	**0.299**	**0.843**	0.74	−2.40	−0.3	0.13	1.01	X
PHD20	1.756	0.709	0.789	0.643	0.787	0.87	−0.31	0.16	0.47	1.27	√
PHD21	1.654	0.815	0.786	0.736	0.769	0.70	−1.28	−0.76	−0.30	0.93	√
PHD22	1.566	0.865	0.785	0.753	0.767	0.72	−1.69	−1.01	−0.48	0.87	√
PSD1	1.387	0.718	0.769	0.66	0.828	1.00	−1.64	−0.29	0.24	0.94	√
PSD2	1.413	0.784	0.735	0.584	0.840	1.00	−1.41	−0.11	0.32	2.03	√
PSD3	1.370	0.641	0.761	0.626	0.833	1.43	−1.34	−0.16	0.15	1.49	√
PSD4	1.264	0.679	0.750	0.704	0.822	1.76	−1.60	−0.63	−0.26	0.85	√
PSD5	1.237	**0.407**	**0.565**	0.474	0.854	1.85	−1.35	−0.73	−0.35	0.87	X
PSD6	1.319	0.566	0.697	0.592	0.838	1.58	−1.38	−0.12	0.23	1.65	√
PSD7	1.230	0.595	0.706	0.696	0.824	1.80	−1.37	−0.63	−0.18	1.15	√
PSD8	1.317	0.652	**0.511**	**0.439**	**0.902**	0.99	−2.09	−1.10	−0.60	1.06	X
PSD9	1.022	0.727	0.614	0.502	**0.893**	1.11	**−3.83**	−1.93	−1.30	−0.05	X
PSD10	1.183	0.648	0.692	0.695	0.876	1.71	−1.83	−0.85	−0.60	0.65	√
PSD11	1.106	0.600	0.640	0.651	0.881	1.68	−2.15	−1.15	−0.62	0.70	√
PSD12	1.198	0.816	0.853	0.795	0.866	2.33	−1.51	−0.76	−0.19	0.83	√
PSD13	1.271	0.791	0.778	0.755	0.870	2.90	−1.32	−0.30	0.05	0.87	√
PSD14	1.288	0.845	0.810	0.777	0.868	2.43	−1.57	−0.60	−0.18	0.48	√
PSD15	1.200	0.820	0.776	0.766	0.869	2.80	−1.45	−0.72	−0.17	0.58	√
PSD16	1.128	0.551	0.724	0.609	0.645	1.91	−2.35	−0.99	−0.35	0.50	√
PSD17	1.215	0.589	0.706	0.632	0.631	1.86	−1.71	−0.87	−0.44	0.62	√
PSD18	1.321	0.441	0.721	0.472	0.695	1.13	−2.43	−0.77	−0.30	0.85	√
PSD19	1.222	0.515	0.655	0.485	0.689	1.61	−1.60	−1.02	−0.59	0.60	√
PSD20	1.310	**0.325**	**0.528**	**0.305**	**0.759**	0.73	**−3.44**	−1.53	−1.08	0.63	X
SOD1	1.403	0.658	**0.578**	**0.382**	**0.776**	0.72	−1.68	−0.04	1.14	2.59	X
SOD2	1.495	**0.221**	**0.594**	**0.327**	**0.797**	0.86	−1.86	−0.71	0.10	1.06	X
SOD3	1.502	0.632	0.761	0.580	0.709	1.07	−1.58	−0.34	0.35	1.04	√
SOD4	1.384	0.728	0.869	0.751	0.649	1.56	−1.47	−0.36	0.16	0.97	√
SOD5	1.365	0.651	0.800	0.701	0.669	1.88	−1.55	−0.45	0.01	0.62	√
SOD6	1.608	0.835	0.867	0.614	0.694	**0.20**	**−4.51**	**−3.13**	−2.34	**3.21**	√
SOD7	1.205	0.695	0.705	0.573	0.718	**0.37**	**−6.67**	**−3.95**	**−3.14**	1.58	√
SOD8	1.589	0.765	0.849	0.612	0.694	**0.27**	**−3.91**	−2.27	−1.66	2.69	√
SOD9	1.114	0.619	0.610	0.526	0.742	0.50	**−5.02**	**−4.03**	−2.72	1.34	√
THD1	**0.961**	**0.362**	0.650	**0.304**	**0.451**	**0.39**	**−8.45**	**−6.33**	**−4.91**	−0.72	X
THD 2	1.348	0.512	0.772	**0.413**	**0.232**	**0.38**	**−6.18**	**−3.55**	−1.78	−0.47	X
THD3	1.438	0.500	0.743	**0.277**	**0.501**	**0.38**	**−5.01**	−0.74	0.64	2.52	X
THD 4	1.265	0.585	0.694	0.495	0.835	**0.37**	**−6.29**	**−3.13**	−1.75	2.30	√
THD 5	1.090	0.741	0.753	0.643	0.807	0.56	**−5.18**	**−3.67**	−1.61	1.13	√
THD 6	1.299	0.732	0.785	0.662	0.801	0.55	**−4.09**	−2.25	−0.90	1.30	√
THD 7	1.440	0.716	0.751	0.574	0.823	**0.33**	**−4.23**	−0.36	1.31	**4.26**	√
THD 8	1.111	0.833	0.804	0.728	0.792	0.45	**−8.11**	**−3.77**	−1.71	1.14	√
THD 9	1.242	0.748	0.745	0.619	0.810	0.62	**−3.61**	−2.49	−1.06	1.23	√

Note: CTT, classical test theory; IRT, item response theory; SD, Standard Deviation; CITC, corrected item-total correlation; CAID, Cronbach’s α if item deleted; PHD, physical domain; PSD, psychological domain; SOD, social domain; THD, therapeutic domain; "√" decision to retain the selected item, "X" to delete the item; boldface indicates values that did not meet standard.

**Table 5:** **Results of the second item-selection phase using CTT and IRT**

**Item**	**SD**	**Factor loading**	**Correlation coefficient**	**CITC**	**CAID**	**IRT**					**Outcome**
						***α***	***b***_***1***_	***b***_***2***_	***b***_***3***_	***b***_***4***_	
PHD1	1.526	0.745	0.685	0.527	0.777	0.54	−2.65	−1.22	−0.31	1.40	√
PHD2	1.467	0.805	0.638	0.473	0.786	0.68	−2.59	−1.11	−0.36	1.06	√
PHD3	1.543	0.643	0.658	0.488	0.785	0.88	−0.94	−0.06	0.71	1.80	√
PHD4	1.350	0.659	0.687	0.551	0.771	1.14	−2.08	−1.18	−0.62	0.22	√
PHD5	1.230	0.828	0.726	0.616	0.762	1.30	−2.33	−1.37	−0.73	−0.19	√
PHD6	1.218	0.818	0.671	0.547	0.773	1.00	−2.79	−1.61	−0.72	0.58	√
PHD7	1.293	0.628	0.686	0.557	0.771	1.02	−2.51	−1.45	−0.46	0.44	√
PHD8	1.282	0.784	0.805	0.637	0.710	1.14	−2.23	−1.17	−0.31	0.77	√
PHD9	1.314	0.737	0.812	0.642	0.706	1.27	−1.84	−0.87	−0.12	0.92	√
PHD10	1.230	0.666	0.777	0.602	0.728	1.58	−1.94	−1.06	−0.45	0.33	√
PHD11	1.491	0.560	0.733	0.470	0.677	1.38	−1.33	−0.65	−0.23	0.47	√
PHD12	1.349	0.673	0.759	0.546	0.627	1.53	−1.61	−0.91	−0.33	0.33	√
PHD13	1.390	**0.515**	0.732	0.497	0.783	1.05	−1.84	−0.68	0.00	1.25	√
PHD14	1.305	0.807	0.711	0.481	0.666	0.77	**−3.04**	−1.74	−0.80	0.83	√
PHD15	1.329	0.772	0.739	0.519	0.643	0.93	−2.19	−1.68	−0.98	0.43	√
PHD16	1.553	0.766	0.833	0.744	0.931	1.23	−1.12	−0.63	−0.19	0.66	√
PHD17	1.546	0.865	0.893	0.833	0.915	1.11	−1.17	−0.64	−0.03	0.87	√
PHD18	1.679	0.836	0.873	0.794	0.922	1.29	−0.46	0.04	0.40	1.03	√
PHD19	1.663	0.888	0.913	0.857	0.910	1.27	−0.59	−0.13	0.23	0.97	√
PHD20	1.606	0.896	0.925	0.879	0.906	1.35	−0.77	−0.26	0.13	0.93	√
PSD1	1.461	0.719	0.763	0.597	0.809	0.88	−1.53	−0.37	0.20	1.86	√
PSD2	1.303	0.714	0.754	0.606	0.805	1.12	−2.21	−0.67	−0.03	1.36	√
PSD3	1.271	0.763	0.780	0.649	0.793	1.38	−1.93	−0.85	−0.26	0.99	√
PSD4	1.338	0.703	0.768	0.622	0.800	1.25	−1.54	−0.37	0.21	1.74	√
PSD5	1.296	0.756	0.806	0.684	0.783	1.50	−1.67	−0.59	−0.01	1.18	√
PSD6	1.232	0.635	0.709	0.546	0.849	1.78	−1.74	−0.84	−0.28	0.72	√
PSD7	1.225	**0.507**	0.786	0.656	0.821	1.65	−1.83	−0.91	−0.34	0.79	√
PSD8	1.260	**0.669**	0.843	0.738	0.799	1.88	−1.62	−0.81	−0.24	0.74	√
PSD9	1.302	0.742	0.806	0.676	0.816	1.93	−1.53	−0.53	−0.19	0.78	√
PSD10	1.236	0.767	0.811	0.693	0.811	2.18	−1.66	−0.75	−0.37	0.48	√
PSD11	1.229	0.556	0.808	0.649	0.730	2.07	−1.72	−0.81	−0.27	0.59	√
PSD12	1.264	0.626	0.847	0.709	0.698	2.12	−1.55	−0.73	−0.20	0.62	√
PSD13	1.352	0.647	0.758	0.544	0.783	1.28	−1.93	−0.88	−0.26	0.64	√
PSD14	1.256	0.430	0.748	0.550	0.777	1.76	−1.62	−0.87	−0.39	0.71	√
SOD1	1.486	0.797	0.885	0.745	**0.914**	1.28	−1.41	−0.19	−0.26	1.05	√
SOD2	1.464	0.878	0.943	0.868	0.811	1.55	−1.20	−0.25	0.25	0.96	√
SOD3	1.511	0.825	0.920	0.814	0.857	1.63	−1.13	−0.20	0.19	0.76	√
SOD4	1.560	0.744	0.848	0.688	0.784	**0.22**	**−5.34**	**−3.21**	−2.21	2.39	√
SOD5	1.233	0.833	0.813	0.680	0.788	**0.39**	**−6.31**	**−3.12**	−2.02	1.70	√
SOD6	1.510	0.790	0.852	0.704	0.774	**0.28**	**−4.74**	−2.26	−1.35	2.60	√
SOD7	1.189	0.766	0.766	0.616	0.814	0.44	**−5.74**	**−3.72**	−2.61	1.12	√
THD1	1.026	0.817	0.820	0.736	0.825	0.54	**−6.29**	**−4.54**	−1.95	0.33	√
THD 2	1.140	0.743	0.798	0.692	0.831	0.47	**−6.03**	**−3.89**	−2.11	0.73	√
THD 3	1.252	0.666	0.711	0.549	**0.861**	**0.33**	**−6.00**	−2.44	1.08	**4.05**	X
THD 4	1.080	0.750	0.736	0.614	0.845	0.50	**−6.89**	**−4.28**	−1.91	0.59	√
THD 5	1.022	0.773	0.775	0.674	0.835	0.54	**−6.19**	**−4.46**	−2.26	0.28	√
THD 6	1.151	0.783	0.796	0.687	0.831	0.59	**−5.00**	**−3.19**	−1.31	0.79	√

Note: CTT, classical test theory; IRT, item response theory; SD, Standard Deviation; CITC, corrected item-total correlation; CAID, Cronbach’s α if item deleted ;"√" decision to retain the selected item, "X" to delete the item; boldface indicates values that did not meet standard.

### First item-selection phase based on CTT and IRT

Five statistical methods (within CTT and IRT) were used to select items. Any item recommended for deletion by two or more methods was deleted. All items were deleted or added based on their item selection results, their clinical importance, and other stroke-specific scales (see Table 4).

As seen in Table 4, 12 items were removed; however, PHD8 (*Do you remember what happened two days ago?*) was not deleted, because previously published results indicate that this item is crucial for the assessment of cognition [15]. PSD1 (*Are you more prone to worry since your illness?*) and PSD2 (*Do you get angry easily?*) did not discriminate well, so PSD2 was deleted in accord with the opinion of patients and experts. PSD15 (*Have you felt depressed while in a cheerful atmosphere?*) was deleted because it was not deemed closely relevant to stroke. As a result, 13 items (PHD18, PHD19, PSD2, PSD5, PSD8, PSD9, PSD15, PSD20, SOD1, SOD2, THD1, THD2, THD3; Additional file 1: Appendix 2-1 ) were deleted. All items in the compliance subdomain (THD1, THD2, THD3) were deleted; thus, this subdomain was also deleted [13-15].

Therefore, the initial scale contained 47 items, 10 subdomains, and 4 domains (see Appendices 3–1 and 3–2).

### Revaluation phase based on CTT and IRT

To ensure the reliability and validity of each item included in the initial scale, we revaluated the items in this scale based on a second item selection. The evaluation results suggested that all items were perfect, except for item THD3. Thus, CTT and IRT analyses in the revaluation phase led to deletion of item THD3 (*Are you satisfied with your medical expenses?*) (see Table 5). As a result, the final scale contained 46 items, 10 subdomains, 4 domains (see Tables 6 and 7). This revision of the Stroke-PROM is described in Table 8.

**Table 6:** **Bank of 46 items in the final Stroke-PROM**

**Item**	**Item**
PHD1. Have you felt numbness in your lips or limbs?	PSD4. Do you worry about your condition getting worse?
PHD2. Have you felt any limb abnormalities (such as a burning sensation)?	PSD5. Have you felt upset?
PHD3. Have you felt limb weakness on just the sick side of your body?	PSD6. Have you felt depressed and passionless?
PHD4. Have you had facial paralysis on one side, and saliva dripping from your mouth?	PSD7. Have you felt frustrated, pessimistic, or in despair about your illness?
PHD5. Have you had difficulty swallowing?	PSD8. Have you felt uninterested in things and people around you?
PHD6. Have you experienced gagging while eating or drinking?	PSD9. Do you consider yourself a burden on your family?
PHD7. Do your hands tremble when you reach for or pick up things?	PSD10. Have you felt hopeless?
PHD8. Do you find it very difficult to focus on one thing?	PSD11. Do you not want to associate with others?
PHD9. Do you have trouble remembering the date?	PSD12. Do you come up with excuses to avoid social activities?
PHD10. When you see an object suddenly, do you struggle to bring its name to mind?	PSD13. When others talk about your disease, do you prefer not to discuss it?
PHD11. Do you have difficulty speaking (such as stammering, unclear enunciation, or pauses)?	PSD14. Have you felt unconfident?
PHD 12. Do you need to repeat yourself to others so that they can understand what you mean?	SOD1. Has your illness affected your family life?
PHD13. Do you remember what happened two days ago?	SOD2. Have you reduced contact with your acquaintances and friends due to your illness?
PHD14. Can you understand what others are saying?	SOD3. Have you avoided some social or family activities due to your illness?
PHD15. Can you recall your children’s or parents’ names?	SOD4. Is your family taking care of your daily life needs?
PHD16. Can you twist a door handle to open the door by yourself?	SOD5. Have your relatives and friends expressed concern about your condition?
PHD17. Can you take care of your own daily needs (such as dressing and bathing)?	SOD6. Has your family reminded you to take your medicine?
PHD18. Can you purchase your daily necessities alone (for example, by going shopping)?	SOD7. Does your family understand you?
PHD19. Can you walk *up and down stairs* alone?	THD1. Are you satisfied with the current effects of your treatment?
PHD20. Can you do light housework (such as making your bed)?	THD2. Are you satisfied with the medical treatment service you receive?
PSD1. Are you more prone to worry since your illness?	THD3. Has treatment at this stage had the effect of reducing your symptoms?
PSD2. Do you struggle to be patient with others?	THD4. Would you like to continue to maintain your current treatment schedule?
PSD3. Do you often feel nervous?	THD5. Has your overall confidence improved since you have been receiving treatment?

Note: PHD, physical domain; PSD, psychological domain; SOD, social domain; THD, therapeutic domain.

**Table 7:** **Scale structure of the bank of 46 items of the final Stroke-PROM**

**Domain**	**Subdomain**	**Item**
Physical domain (PHD)	Somatic symptom (SOS)	1-, 2-, 3-, 4-, 5-, 6-, 7-
	Cognition (COG)	8-, 9-, 10-, 13
	Verbal communication (VEC)	11-, 12-, 14, 15
	Self-help skills (SHS)	16, 17, 18, 19, 20
Psychological domain (PSD)	Anxiety (ANX)	1-, 2-, 3-, 4-, 5-
	Depression (DEP)	6-, 7-, 8-, 9-, 10-
	Avoidance (AVO)	11-, 12-, 13-, 14-
Social domain (SOD)	Social contacts (SOC)	1-, 2-, 3-
	Family support (FAS)	4, 5, 6, 7
Therapeutic domain (THD)	Satisfaction (SAT)	1, 2, 3, 4, 5

Note: “-” indicates a reverse-scored item.

**Table 8:** **Process of revising the Stroke-PROM**

	**Formation of preliminary items**	**Formation of preliminary scale**	**Formation of initial scale**	**Formation of final scale**
Number of items	62	60	47	46
Number of domains	4	4	4	4
Number of subdomains	10	11 (cognition subdomain was added)	10 (compliance subdomain was deleted)	10
Number of deleted items	—	7 (PHD3, PHD5, PHD6, PHD9, PHD16, PSD18, SOD1 of Additional file 1: Appendix 1-1)	13 (PHD18, PHD19, PSD2, PSD5, PSD8, PSD9, PSD15, PSD20, SOD1, SOD2, THD1, THD2, THD3 of Additional file 1: Appendix 2-1)	1 (THD3 of Additional file 1: Appendix 3-1)
Method used for deleting items	—	I-CVI (PHD3, PHD5, PHD6, PHD9, SOD1); Opinions of patients and clinical experts, reference to other scales (PHD16, PSD18)	CTT and IRT (PHD18, PHD19, PSD5, PSD8, PSD9, PSD20, SOD1, SOD2, THD1); IRT, opinions of patients and clinical experts, reference to other scales (THD2, THD3); Opinions of patients and clinical experts, reference to other scales (PSD2, PSD15)	CTT and IRT (THD3)
Number of added items	—	5 (PHD2, PHD7, PHD10, PHD11, PSD18 of Additional file 1: Appendix 2-1)	0	0
Method used for adding items	—	Opinions of patients and clinical experts, reference to other scales	—	—

Note: PHD, physical domain; PSD, psychological domain; SOD, social domain; THD, therapeutic domain.

### Evaluation of the scale

The validity, reliability, and responsiveness of the remaining 46 items were assessed and the results are presented in the sections below.

### Content validity

The content validity was achieved as outlined in the Methods and was confirmed using the values obtained for the CVIs (see Table 3 and “Item generation” in the Results).

### Construct validity

We conducted confirmatory factor analysis (CFA) on the 46 Stroke-PROM items. The index of fit (GFI, RMR, NFI, NNFI, CFI, IFI) met the standard requirements (see Table 9).

**Table 9:** **Goodness of fit statistics of the Stroke-PROM**

**Domain**	**GFI**	**RMR**	**NFI**	**NNFI**	**CFI**	**IFI**
PHD	0.79	0.086	0.88	0.88	0.90	0.90
PSD	0.89	0.058	0.95	0.95	0.96	0.96
SOD	0.90	0.092	0.92	0.88	0.93	0.93
THD	0.94	0.043	0.95	0.91	0.96	0.96

Note: PHD, physical domain; PSD, psychological domain; SOD, social domain; THD, therapeutic domain; GFI, goodness-of-fit index; NFI, normed fit index; NNFI, non-normed fit index; IFI, incremental fit index; CFI, comparative fit index; RMR, root mean square residual.

Table 10 presents the 10 subdomains, their corresponding items and standardized factor loadings produced from the CFA. The standardized factor loadings for each of the 46 Stroke-PROM items were above 0.5, except for items PHD1, PHD2, and PHD3; however, these three items were recommended for retention by the results of CTT and IRT analyses. The results indicated that the 46 items showed salient loadings on their specific subdomains, and these 10 subdomains correlated well with the 10 that were conceptualized in the design phase and indicated good construct validity.

**Table 10:** **Maximum likelihood estimation of CFA for the Stroke-PROM**

**Subdomain**	**Item**	**Non-standardized factor loading**	**Standard error**	***t***	***R*** ^***2***^	**Non-standardized factor loading error var**	**Standardized factor loading**	**Standardized factor loading error var**
SOS	PHD1	0.67	0.07	9.40	0.20	1.87	0.44	0.80
	PHD2	0.56	0.07	8.05	0.15	1.83	0.38	0.85
	PHD3	0.67	0.07	9.25	0.19	1.93	0.44	0.81
	PHD4	0.89	0.06	15.17	0.44	1.02	0.66	0.56
	PHD5	1.01	0.05	20.25	0.67	0.50	0.82	0.33
	PHD6	0.93	0.05	18.25	0.58	0.62	0.76	0.42
	PHD7	0.84	0.06	14.91	0.43	0.96	0.65	0.57
COG	PHD8	0.93	0.06	16.79	0.53	0.77	0.73	0.47
	PHD9	0.94	0.06	16.50	0.51	0.84	0.72	0.49
	PHD10	0.92	0.05	17.38	0.56	0.67	0.75	0.44
VEC	PHD11	1.02	0.07	15.41	0.47	1.18	0.68	0.53
	PHD12	1.02	0.06	17.35	0.57	0.79	0.75	0.43
COG	PHD13	0.82	0.06	12.96	0.35	1.25	0.59	0.65
VEC	PHD14	0.65	0.06	10.49	0.25	1.28	0.50	0.75
	PHD15	0.71	0.06	11.37	0.28	1.27	0.53	0.72
SHS	PHD16	1.17	0.06	19.09	0.57	1.04	0.76	0.43
	PHD17	1.30	0.06	22.31	0.70	0.71	0.84	0.30
	PHD18	1.40	0.06	22.10	0.69	0.86	0.83	0.31
	PHD19	1.52	0.06	25.72	0.84	0.45	0.91	0.16
	PHD20	1.50	0.06	26.74	0.87	0.33	0.93	0.13
ANX	PSD1	0.90	0.06	14.01	0.38	1.32	0.62	0.62
	PSD2	0.83	0.06	14.45	0.40	1.02	0.63	0.60
	PSD3	0.92	0.05	17.14	0.52	0.77	0.72	0.48
	PSD4	0.99	0.06	17.74	0.55	0.81	0.74	0.45
	PSD5	1.04	0.05	19.85	0.64	0.60	0.80	0.36
DEP	PSD6	0.79	0.05	14.88	0.41	0.89	0.64	0.59
	PSD7	0.87	0.05	17.05	0.51	0.74	0.71	0.49
	PSD8	1.02	0.05	20.57	0.66	0.54	0.81	0.34
	PSD9	0.96	0.05	18.02	0.55	0.77	0.74	0.45
	PSD10	0.95	0.05	18.88	0.59	0.63	0.77	0.41
AVO	PSD11	0.97	0.05	19.25	0.62	0.57	0.79	0.38
	PSD12	1.04	0.05	20.31	0.67	0.53	0.82	0.33
	PSD13	0.81	0.06	13.53	0.36	1.17	0.60	0.64
	PSD14	0.80	0.06	14.56	0.41	0.93	0.64	0.59
SOC	SOD1	1.16	0.06	19.83	0.61	0.86	0.78	0.39
	SOD2	1.40	0.05	26.97	0.92	0.17	0.96	0.08
	SOD3	1.33	0.06	23.42	0.77	0.53	0.88	0.23
FAS	SOD4	1.27	0.06	19.81	0.66	0.82	0.81	0.34
	SOD5	0.88	0.05	16.63	0.51	0.75	0.71	0.49
	SOD6	1.23	0.06	19.83	0.66	0.77	0.81	0.34
	SOD7	0.77	0.05	14.67	0.42	0.82	0.65	0.58
SAT	THD1	0.83	0.04	20.28	0.66	0.36	0.81	0.34
	THD2	0.81	0.05	16.69	0.50	0.65	0.71	0.50
	THD4	0.78	0.05	17.08	0.52	0.56	0.72	0.48
	THD5	0.77	0.04	18.22	0.57	0.45	0.75	0.43
	THD6	0.85	0.05	17.71	0.55	0.60	0.74	0.45

Note: PHD, physical domain; PSD, psychological domain; SOD, social domain; THD, therapeutic domain; SOS, somatic symptom; COG, cognition; VEC, verbal communication; SHS, self-help skills; ANX, anxiety; DEP, depression; AVO, avoidance; SOC, social contacts; FAS, family support; COM, compliance; SAT, satisfaction.

### Reliability

Cronbach’s α coefficient ≥0.70 is considered acceptable for internal consistency. Cronbach’s α coefficient was 0.905 for the total score, and for the four domains, it ranged from 0.861 to 0.908. These results indicated high internal consistency (see Table 11).

**Table 11:** **Cronbach’s α coefficient of four domains and total scale**

**Domain**	**Cronbach’s α coefficient**
Physical	0.888
Psychological	0.908
Social	0.879
Therapeutic	0.861
Total	0.905

### Discriminant validity

The discriminant validity of each subdomain was examined by comparing mean scores across healthy participants and the groups of stroke patients as defined by their modified Rankin scores. Table 12 indicates that the scales for 9 of the 10 subdomains were significantly different across healthy participants and stroke patients with different degrees of disability and dependence as defined by the modified Rankin scale. Because healthy participants were not treated and therefore could not answer the items in the treatment domain, no comparison of healthy participants was made for the SAT subdomain. However, the SAT subdomain scores for the stroke patient population was not significantly different across the Rankin levels. Overall, the Stroke-PROM was able to differentiate between healthy participants and stroke patients with varying degrees of disability and dependence as defined by the modified Rankin scale.

**Table 12:** **Subdomain scores obtained using the Stroke-PROM instrument in healthy controls and stroke patients with varying degrees of disability and dependence as defined by the modified Rankin scale (mean ± SD)**

**Sub-domain**	**Control**	**Rankin level**						***F***	***P***
		**0**	**1**	**2**	**3**	**4**	**5**		
SOS	32.95 ± 3.02	27.90 ± 6.57	26.31 ± 6.20	24.85 ± 6.30	24.53 ± 6.35	25.98 ± 4.96	23.46 ± 6.96	25.652	<0.001
COG	19.33 ± 2.27	16.59 ± 3.80	15.69 ± 3.78	13.32 ± 3.32	13.33 ± 3.38	13.86 ± 3.31	13.42 ± 4.79	32.806	<0.001
VEC	19.43 ± 1.98	17.96 ± 3.09	16.45 ± 3.26	14.22 ± 2.82	12.54 ± 3.37	13.81 ± 3.00	12.62 ± 4.51	59.779	<0.001
SHS	23.61 ± 3.03	24.33 ± 1.59	19.39 ± 3.16	15.68 ± 2.03	12.19 ± 2.33	8.91 ± 1.94	5.59 ± 1.26	769.629	<0.001
ANX	22.85 ± 2.90	17.61 ± 5.03	17.55 ± 5.31	17.34 ± 4.31	16.88 ± 4.51	16.07 ± 5.17	15.93 ± 5.86	21.911	<0.001
DEP	24.11 ± 2.11	21.04 ± 4.26	19.09 ± 4.71	18.44 ± 4.30	17.83 ± 4.90	17.45 ± 5.13	17.87 ± 5.71	25.407	<0.001
AVO	19.36 ± 1.75	16.79 ± 3.38	15.29 ± 3.89	14.00 ± 3.89	14.10 ± 3.43	14.26 ± 4.02	14.70 ± 4.97	24.756	<0.001
SOC	14.49 ± 1.55	11.88 ± 3.87	9.03 ± 4.09	9.63 ± 2.95	9.95 ± 3.61	9.48 ± 3.70	8.72 ± 4.43	31.575	<0.001
FAS	8.76 ± 4.72	14.27 ± 4.82	14. 90 ± 3.95	15.12 ± 4.95	14.33 ± 4.35	15.48 ± 4.49	16.25 ± 4.56	28.217	<0.001
SAT	--	24.19 ± 4.83	24.30 ± 4.32	22.90 ± 6.23	23.35 ± 5.61	21.88 ± 5.99	24.03 ± 5.40	1.929	>0.050
*n*	104	135	123	41	58	42	76		

Note: SOS, somatic symptom; COG, cognition; VEC, verbal communication; SHS, self-help skills; ANX, anxiety; DEP, depression; AVO, avoidance; SOC, social contacts; FAS, family support; SAT, satisfaction.

### Feasibility

Both the response rate and the completion rate of the Stroke-PROM tool were more than 97%. The average completion time was 8.9 minutes.

## Discussion

In this study, we developed and validated a Stroke-PROM for use in the evaluation of outcomes for patients with stroke. The US Food and Drug Administration (FDA) has highlighted the importance of the use of PRO in clinical trials and provided guidance regarding the development of PROMs [24]. The development strategy for the Stroke-PROM in this study complied with those guidelines. To the best of our knowledge, this is the first Stroke-PROM specifically developed and validated for use in clinical trials of new drugs with stroke to include physical, psychological, social and therapeutic domains [25].

The most commonly used stroke-specific measures, the National Institutes of Health Stroke Scale and the Canadian Neurological Scale are clinician-reported outcome measures that assess only the physical aspects of stroke [26-28]. Although the Stroke Impact Scale Version 2.0, the Stroke and Aphasia Quality of Life Scale-39, the Newcastle Stroke-Specific Quality of Life Measure, and the Stroke-Specific Quality of Life measures are all multidimensional PRO measures, no measures have been developed that assess the subdomains of family support and patient satisfaction with treatment [13-15,29]. In contrast to other stroke-specific instruments, our instrument includes these subdomains and therefore fills a gap in the research arena for a stroke PROM [13-15,29-31].

Stroke has considerable adverse physical and psychological impacts on patients over time. Stroke patients need help, understanding and care from their families [32]. Indeed, a growing body of research demonstrates the importance of family relationships for the recovery of functional capacity after stroke [33,34]. A stroke survivor’s family is often the most important source of long-term support during the patients’ recovery and treatment, and family support plays a significant role throughout the poststroke recovery period [35-38]. Family can supply the stroke survivor with physical and mental support, such as providing care in daily life and understanding [39]. Therefore, family support is a necessary addition to the Stroke-PROM.

Satisfaction with treatment is a main outcome measure in new drug clinical trials [24,40]. A Stroke-PROM tool can be used to measure treatment benefit or risk during clinical trials for medical products. Additionally, Stroke-PROM instruments provide optimal information from the patient’s perspective for use in drawing conclusions about the effectiveness of treatment [24]. Thus, the inclusion of a subdomain for treatment satisfaction provides an opportunity for new drug clinical trial participants to integrate into the overall evaluation the different aspects of their responses to treatment, including pain relief, function improvement, and side effects, as well as to provide feedback about the potential acceptability of a new drug and their overall trust in the drug treatment [24,41,42]. Therefore, the subdomain of satisfaction with treatment is also a prudent addition to the Stroke-PROM.

The Stroke-PROM presented here would complement existing stroke-specific measures and has particular value for extending our understanding of the impact of family support and patient satisfaction with treatment in clinical trials of new drugs for stroke. During clinical trials, the Stroke-PROM can be used to simultaneously measure the effect of a medical intervention on several concepts, that is, the measured parameter, such as a symptom or group of symptoms, the medical intervention effects on a particular function or group of functions, or a group of symptoms or functions shown to measure the severity of a health condition. The use of the Stroke-PROM as an outcome measure in clinical trials may facilitate evaluation of the effectiveness across several therapeutic modalities. From the researcher’s perspective, the scale may capture the patient’s experience and treatment benefit or risk, assist researchers in determining which patients with stroke benefit meaningfully from treatment, and facilitate between-trial comparisons [24]. From the pharmaceutical company’s perspective, such an instrument may increase the efficiency of discussions with the FDA during the medical product development process, and provide optimal information from a patient’s perspective for use in making conclusions about treatment effects at the time of medical product approval [24]. From a regulatory perspective, the Stroke-PROM tool may provide a standardized method for assessing treatment effectiveness on basic symptoms so that claims can be supported with PRO evidence in medical product clinical trials [43].

In contrast to the development of other stroke-specific instruments, our study used I-CVI, IRT and CFA as rigorous evidence for item selection, validity and reliability. First, content validity is an essential step in the development of any new scale. None of the previously developed instruments for stroke used statistical methods such as CVI to quantify content validity as was done for the Stroke-PROM tool in our study. The FDA places particular emphasis on demonstrating content validity using open-ended interviews with patients [24]. Identifying the items of the Stroke-PROM based on a review of the literature and other stroke questionnaires, face-to-face interviews with patients, discussions with stroke professionals and the CVI further strengthened the content validity of the preliminary scale in our study.

Second, in the item-selection phase, analyses based on both CTT and IRT were used to delete items. The IRT-based analysis was used more heavily than that based on CTT in the construction of scales for measuring subjective attributes. IRT-based analysis also afforded more accurate examinations of the features of each scale item than the analyses based on CTT. Existing stroke-specific instruments had focused exclusively on CTT statistics (e.g., exploratory factor analysis, Cronbach’s α coefficient) [13,29]. No other stroke-specific instruments existed that had been developed using IRT. CTT statistics are associated with certain disadvantages, whereas methods based on IRT offer several advantages to refine items and therefore to improve on CTT [44].

In our study, both CTT and IRT analyses were repeated during finalization of the item content in the second sample. The results showed that the final Stroke-PROM had a high degree of reliability and validity.

Third, the instrument’s presumed internal structure, supported by CFA, confirmed that the Stroke-PROM measure is multidimensional in nature. No other stroke-specific instruments have established construct validity with CFA.

In summary, our results showed that the scale was valid, reliable and feasible and had strong discriminative properties between healthy controls and stroke patients with varying degrees of disability and dependence as defined by the modified Rankin scale. Although the Stroke-PROM tool was developed primarily for use in clinical trials of new drugs to evaluate their clinical therapeutic effects, this study showed that the Stroke-PROM also had strong discriminative measurement properties and could be used to differentiate patients with stroke from healthy controls. We therefore believe that there is an important role for this Stroke-PROM instrument in clinical practice as well as in clinical trials.

### Limitations and further development

The scale has several potential limitations that we will address in future studies.

First, in the evaluation of validity, our study did not explicitly address criterion validity. Most of the patients with stroke were elderly, and completion of two or more scales would have been a significant burden for them, according to our experts on stroke. Stroke patients often experience disturbances of consciousness and physical restlessness; thus, adding more tests could produce test fatigue in these patients, thereby reducing the validity and reliability of measurement. Therefore, instead of asking patients to complete more than one scale, we chose to delay a thorough examination of criterion validity for a future investigation.

Second, test–retest reliability was not measured as part of the validation process. This was due in part to the additional burden it would have placed on patients, but also because of the difficulties inherent in follow-up with patients in their home communities and rural areas. We therefore demonstrated reliability only with internal consistency; however, we conducted our reliability evaluation of items at two points in the process: during the phases of item selection and scale evaluation.

Third, the stroke patient sample differed slightly from the healthy participant sample in two ways: the stroke patient population had a higher proportion of males and of individuals over 65 years old. Future studies should seek to balance these groups.

Because of limited resources (both funding and personnel), the sample populations may not be representative of the entire population of patients with stroke. Our participants were from only the Shanxi province in northern China. Thus, future studies should evaluate reliability and validity of the Stroke-PROM instrument with a nationwide sample.

The Stroke-PROM was administered to native-Chinese-speaking individuals. Therefore, further work is required to test the strengths and weaknesses of this instrument across various national, cultural and language contexts.

## Conclusions

Our results provided evidence for satisfactory reliability and validity of the Stroke-PROM. However, the instrument will require additional revisions and improvements through testing in different populations. The ongoing process of modifying the Stroke-PROM will also encompass further validation and reliability testing across various applications of the instrument. The Stroke-PROM is not meant to replace existing stroke-specific measures, but to provide further valuable information on patients with stroke. This innovative instrument may be helpful in both routine medical practice and clinical research.

## Additional file

Additional file 1:
**Appendix 1–1**. Bank of 62 preliminary items of the Stroke-PROM **Appendix 1–2**. Scale structure of the bank of 62 preliminary items of the Stroke-PROM. **Appendix 2–1**. Bank of 60 items of the preliminary Stroke-PROM. **Appendix 2–2**. Scale structure of the bank of 60 items of the preliminary Stroke-PROM. **Appendix 3–1**. Bank of 47 items of the initial Stroke-PROM. **Appendix 3–2**. Scale structure of the bank of 47 items of the initial Stroke-PROM.

## Abbreviations

PRO: Patient-reported outcomes
PROM: Patient-reported outcomes measure
HRQOL: Health-related quality of life
EQ-5D: EuroQol 5 dimension
SF-36: Short Form 36
SF-12: The short form 12
SIP: The sickness impact profile
NEWSQOL: Newcastle stroke-specific quality of life measure
NIHSS: National institutes of health stroke scale
SAQOL-39: Stroke and aphasia quality of life scale - 39 item version
SIS: Stroke impact scale & stroke toolbox
SS-QOL: Stroke-specific quality of life measure
CNS: Canadian neurological scale
mRS-SI: Structured interview for the modified rankin scale
CTT: Classical test theory
SD: Standard deviation
CAID: Cronbach’s α if item deleted
IRT: item response theory
CITC: Corrected item-total correlation
CVI: Content validity index
I-CVI: Item-level CVI
CFA: Confirmatory factor analysis
KMO: Kaiser-meyer-olkin
GFI: Goodness of fit index
NFI: Normed fit index
NNFI: Non normed fit index
IFI: Incremental fit index
CFI: Comparative fit index
RMR: Root mean square residual
PHD: Physical domain
PSD: Psychological domain
SOD: Social domain
THD: Therapeutic domain
SOS: Somatic symptom
COG: Cognition
VEC: Verbal communication
SHS: Self-help skills
ANX: Anxiety
DEP: Depressed
AVO: Avoid
SOC: Social contacts
FAS: Family support
COM: Compliance
SAT: Satisfaction
FDA: Food and drug administration
