# Supplementary material for: Development and validation of a patient-reported outcome measure for stroke patients
Source: Health Qual Life Outcomes. 2015 May 8;13:53. doi: 10.1186/s12955-015-0246-0 (PMC4489208; doi:10.1186/s12955-015-0246-0)
Supplement: Additional file 1: — Appendix 1–1. Bank of 62 preliminary items of the Stroke-PROM Appendix 1–2. Scale structure of the bank of 62 preliminary items of the Stroke-PROM. Appendix 2–1. Bank of 60 items of the preliminary Stroke-PROM. Appendix 2–2. Scale structure of the bank of 60 items of the preliminary Stroke-PROM. Appendix 3–1. Bank of 47 items of the initial Stroke-PROM. Appendix 3–2. Scale structure of the bank of 47 items of the initial Stroke-PROM. [file 12955_2015_246_MOESM1_ESM.doc]

**Appendix 1-1. Bank of 62 preliminary items of the Stroke-PROM**

| **item** | **item** |
| --- | --- |
| PHD1. Have you felt numbness in your lips, tongue, or limbs? | PSD9. Do you have bouts of crying or feeling like you want to cry? |
| PHD2. Have you had facial paralysis on one side, and saliva dripping from your mouth? | PSD10. Have you felt depressed and passionless? |
| PHD3. Have you experienced headaches or dizziness? | PSD11. Have you felt frustrated, pessimistic, or in despair about your illness? |
| PHD4. Have you felt limb weakness on just the sick side of your body? | PSD12. Have you felt uninterested in things and people around you? |
| PHD5. Has your hearing seemed to decline recently? | PSD13. Do you consider yourself a burden on your family? |
| PHD6. Has your vision been blurry at times recently? | PSD14. Have you felt hopeless? |
| PHD7. Have you had difficulty swallowing? | PSD15. Have you felt depressed while in a cheerful atmosphere? |
| PHD8. Have you experienced gagging while eating or drinking? | PSD16. Do you not want to associate with others? |
| PHD9. Do you tire easily? | PSD17. Do you come up with excuses to avoid social activities? |
| PHD10. Do you remember what happened two days ago? | PSD18. Have you felt uncomfortable around unfamiliar people? |
| PHD11. Do you find it very difficult to focus on one thing? | PSD19. Have you felt unconfident? |
| PHD 12. Do you have difficulty speaking? | PSD20. Have you spent time reflecting on the past? |
| PHD13. Do you need to repeat yourself to others so that they can understand what you mean? | SOD1. Do you love to brag about your own abilities and your impressive past? |
| PHD14. Can you understand what others are saying? | SOD2. Do you have enough energy to do what you want to do? |
| PHD15. Can you recall your children’s or parents’ names? | SOD3. Have you given up some of your old hobbies because of your illness? |
| PHD16. Are you able to talk to others on the phone? | SOD4. Has your illness affected your family life? |
| PHD17. Can you twist a door handle to open the door by yourself? | SOD5. Have you reduced contact with your acquaintances and friends due to your illness? |
| PHD18. Can you take care of your own daily needs? | SOD6. Have you avoided some social or family activities due to your illness? |
| PHD19. Do you have difficulty standing up from the sofa? | SOD7. Is your family taking care of your daily life needs? |
| PHD20. Can you purchase your daily necessities alone? | SOD8. Have your relatives and friends expressed concern about your condition? |
| PHD21. Can you walk up and down stairs alone? | SOD9. Have your family reminded you to take your medicine? |
| PHD22. Can you do light housework? | SOD10. Does your family understand you? |
| PHD23. Are you incontinent? | THD1. **Have you taken your medications regularly** according to your doctor's orders ? |
| PSD1. Are you more prone to worry since your illness? | THD2. **Have you** changed bad habits according to your doctor’s orders? |
| PSD2. Do you get angry easily? | THD3. **Have you** regularly kept medical appointments according to your doctor’s orders? |
| PSD3. Do you struggle to be patient with others? | THD4. **Has** treatment at this stage had the effect of reducing your symptoms? |
| PSD4. Do you feel nervous? | THD5. Are you satisfied with the current effects of your treatment? |
| PSD5. Are you unable to concentrate on one thing? | THD6. Are you satisfied with the medical treatment service you receive? |
| PSD6. Do you worry about your condition getting worse? | THD7. Are you satisfied with your medical expenses? |
| PSD7. Have you been alone and felt and upset? | THD8. Would you like to continue to maintain your current treatment schedule? |
| PSD8. Do you feel tired and bored, and not want to do anything? | THD9. Has your overall confidence improved since you have been receiving treatment? |

Note: PHD, physical domain; PSD, psychological domain; SOD, social domain; THD, therapeutic domain.

**Appendix 1-2. Scale structure of the bank of 62 preliminary items of the Stroke-PROM**

| **Domain** | **Subdomain** | **Item** |
| --- | --- | --- |
| Physical domain (PHD) | Somatic symptom (SOS) | 1-, 2-, 3-, 4-, 5-, 6-, 7- |
|  |  | 8-, 9-, 10, 11- |
|  | Verbal communication (VEC) | 12-, 13-, 14, 15, 16 |
|  | Self-help skills (SHS) | 17, 18, 19-, 20, 21, 22, 23- |
| Psychological domain (PSD) | Anxiety (ANX) | 1-, 2-, 3-, 4-, 5-, 6-, 7- |
|  | Depression (DEP) | 8-, 9-, 10-, 11-, 12-, 13-, 14-, 15- |
|  | Avoidance (AVO) | 16-, 17-, 18-, 19-, 20- |
| Social domain (SOD) | Social contacts (SOC) | 1-, 2, 3-, 4-, 5-, 6- |
|  | Family support (FAS) | 7, 8, 9, 10 |
| Therapeutic domain (THD) | Compliance (COM) | 1, 2, 3 |
|  | Satisfaction (SAT) | 4, 5, 6, 7, 8, 9 |

Note: "-" indicates a reverse-scored item.

**Appendix 2-1. Bank of 60** **items of the preliminary Stroke-PROM**

| **item** | **item** |
| --- | --- |
| PHD1. Have you felt numbness in your lips or limbs? | PSD9. Do you have bouts of crying or feeling like you want to cry? |
| PHD2. Have you felt any limb abnormalities (such as a burning sensation)? | PSD10. Have you felt depressed and passionless? |
| PHD3. Have you had facial paralysis on one side, and saliva dripping from your mouth? | PSD11. Have you felt frustrated, pessimistic, or in despair about your illness? |
| PHD4. Have you felt limb weakness on just the sick side of your body? | PSD12. Have you felt uninterested in things and people around you? |
| PHD5. Have you had difficulty swallowing? | PSD13. Do you consider yourself a burden on your family? |
| PHD6. Have you experienced gagging while eating or drinking? | PSD14. Have you felt hopeless? |
| PHD7. Do your hands tremble when you reach for or pick up things? | PSD15. Have you felt depressed while in a cheerful atmosphere? |
| PHD8. Do you remember what happened two days ago? | PSD16. Do you not want to associate with others? |
| PHD9. Do you find it very difficult to focus on one thing? | PSD17. Do you come up with excuses to avoid social activities? |
| PHD10. Do you have trouble remembering the date? | PSD18. When others talk about your disease, do you prefer not to discuss it? |
| PHD11. When you see an object suddenly, do you struggle to bring its name to mind? | PSD19. Have you felt unconfident? |
| PHD 12. Do you have difficulty speaking (such as stammering, unclear enunciation, or pauses)? | PSD20. Have you spent time reflecting on the past? |
| PHD13. Do you need to repeat yourself to others so that they can understand what you mean? | SOD1. Do you have enough energy to do what you want to do? |
| PHD14. Can you understand what others are saying? | SOD2. Have you given up some of your old hobbies because of your illness? |
| PHD15. Can you recall your children’s or parents’ names? | SOD3. Has your illness affected your family life? |
| PHD16. Can you twist a door handle to open the door by yourself? | SOD4. Have you reduced contact with your acquaintances and friends due to your illness? |
| PHD17. Can you take care of your own daily needs (such as dressing and bathing)? | SOD5. Have you avoided some social or family activities due to your illness? |
| PHD18. Are you incontinent? | SOD6. Is your family taking care of your daily life needs? |
| PHD19. Do you have difficulty standing up from the sofa? | SOD7. Have your relatives and friends expressed concern about your condition? |
| PHD20. Can you purchase your daily necessities alone (for example, by going shopping)? | SOD8. Have your family reminded you to take your medicine? |
| PHD21. Can you walk up and down stairs alone? | SOD9. Does your family understand you? |
| PHD22. Can you do light housework (such as making your bed)? | THD1. **Have you taken your medications regularly** according to your doctor's orders ? |
| PSD1. Are you more prone to worry since your illness? | THD2. **Have you** changed bad habits according to your doctor’s orders? |
| PSD2. Do you get angry easily? | THD3. **Have you** regularly kept medical appointments according to your doctor’s orders? |
| PSD3. Do you struggle to be patient with others? | THD4. **Has** treatment at this stage had the effect of reducing your symptoms? |
| PSD4. Do you feel nervous? | THD5. Are you satisfied with the current effects of your treatment? |
| PSD5. Are you unable to concentrate on one thing? | THD6. Are you satisfied with the medical treatment service you receive? |
| PSD6. Do you worry about your condition getting worse? | THD7. Are you satisfied with your medical expenses? |
| PSD7. Have you felt upset? | THD8. Would you like to continue to maintain your current treatment schedule? |
| PSD8. Do you feel tired and bored, and not want to do anything? | THD9. Has your overall confidence improved since you have been receiving treatment? |

Note: PHD, physical domain; PSD, psychological domain; SOD, social domain; THD, therapeutic domain.

**Appendix 2-2.** **Scale structure of the bank of 60 items of the preliminary Stroke-PROM**

| **Domain** | **Subdomain** | **item** |
| --- | --- | --- |
| Physical domain (PHD) | Somatic symptom (SOS) | 1-, 2-, 3-, 4-, 5-, 6-, 7- |
|  | Cognition (COG) | 8, 9-, 10-, 11- |
|  | Verbal communication (VEC) | 12-, 13-, 14, 15 |
|  | Self-help skills (SHS) | 16, 17, 18-, 19-, 20, 21, 22 |
| Psychological domain (PSD) | Anxiety (ANX) | 1-, 2-, 3-, 4-, 5-, 6-, 7- |
|  | Depression (DEP) | 8-, 9-, 10-, 11-, 12-, 13-, 14-, 15- |
|  | Avoidance (AVO) | 16-, 17-, 18-, 19-, 20- |
| Social domain (SOD) | Social contacts (SOC) | 1, 2-, 3-, 4-, 5- |
|  | Family support (FAS) | 6, 7, 8, 9 |
| Therapeutic domain (THD) | Compliance (COM) | 1, 2, 3 |
|  | Satisfaction (SAT) | 4, 5, 6, 7, 8, 9 |

Note: "-" indicates a reverse-scored item.

**Appendix 3-1. Bank of 47 items of the initial Stroke-PROM**

| **item** | **item** |
| --- | --- |
| PHD1. Have you felt numbness in your lips or limbs? | PSD5. Have you felt upset? |
| PHD2. Have you felt any limb abnormalities (such as a burning sensation)? | PSD6. Have you felt depressed and passionless? |
| PHD3. Have you felt limb weakness on just the sick side of your body? | PSD7. Have you felt frustrated, pessimistic, or in despair about your illness? |
| PHD4. Have you had facial paralysis on one side, and saliva dripping from your mouth? | PSD8. Have you felt uninterested in things and people around you? |
| PHD5. Have you had difficulty swallowing? | PSD9. Do you consider yourself a burden on your family? |
| PHD6. Have you experienced gagging while eating or drinking? | PSD10. Have you felt hopeless? |
| PHD7. Do your hands tremble when you reach for or pick up things? | PSD11. Do you not want to associate with others? |
| PHD8. Do you find it very difficult to focus on one thing? | PSD12. Do you come up with excuses to avoid social activities? |
| PHD9. Do you have trouble remembering the date? | PSD13. When others talk about your disease, do you prefer not to discuss it? |
| PHD10. When you see an object suddenly, do you struggle to bring its name to mind? | PSD14. Have you felt unconfident? |
| PHD11. Do you have difficulty speaking (such as stammering, unclear enunciation, or pauses)? | SOD1. Has your illness affected your family life? |
| PHD 12. Do you need to repeat yourself to others so that they can understand what you mean? | SOD2. Have you reduced contact with your acquaintances and friends due to your illness? |
| PHD13. Do you remember what happened two days ago? | SOD3. Have you avoided some social or family activities due to your illness? |
| PHD14. Can you understand what others are saying? | SOD4. Is your family taking care of your daily life needs? |
| PHD15. Can you recall your children’s or parents’ names? | SOD5. Have your relatives and friends expressed concern about your condition? |
| PHD16. Can you twist a door handle to open the door by yourself? | SOD6. Have your family reminded you to take your medicine? |
| PHD17. Can you take care of your own daily needs (such as dressing and bathing)? | SOD7. Does your family understand you? |
| PHD18. Can you purchase your daily necessities alone (for example, by going shopping)? | THD1. Are you satisfied with the current effects of your treatment? |
| PHD19. Can you walk up and down stairs alone? | THD2. Are you satisfied with the medical treatment service you receive? |
| PHD20. Can you do light housework (such as making your bed)? | THD3. Are you satisfied with your medical expenses? |
| PSD1. Are you more prone to worry since your illness? | THD4. **Has** treatment at this stage had the effect of reducing your symptoms? |
| PSD2. Do you struggle to be patient with others? | THD5. Would you like to continue to maintain your current treatment schedule? |
| PSD3. Do you feel nervous? | THD6. Has your overall confidence improved since you have been receiving treatment? |
| PSD4. Do you worry about your condition getting worse? |  |

Note: PHD, physical domain; PSD, psychological domain; SOD, social domain; THD, therapeutic domain.

**Appendix 3-2. Scale structure of the bank of 47 items of the initial Stroke-PROM**

| **Domain** | **Subdomain** | **Item** |
| --- | --- | --- |
| Physical domain (PHD) | Somatic symptom (SOS) | 1-, 2-, 3-, 4-, 5-, 6-, 7- |
|  | Cognition (COG) | 8-, 9-, 10-, 13 |
|  | Verbal communication (VEC) | 11-, 12-, 14, 15 |
|  | Self-help skills (SHS) | 16, 17, 18, 19, 20 |
| Psychological domain (PSD) | Anxiety (ANX) | 1-, 2-, 3-, 4-, 5- |
|  | Depression (DEP) | 6-, 7-, 8-, 9-, 10- |
|  | Avoidance (AVO) | 11-, 12-, 13-, 14- |
| Social domain (SOD) | Social contacts (SOC) | 1-, 2-, 3- |
|  | Family support (FAS) | 4, 5, 6, 7 |
| Therapeutic domain (THD) | Satisfaction (SAT) | 1, 2, 3, 4, 5, 6 |

Note: "-" indicates a reverse-scored item.
